# Diversity of *Trichoderma* in the unexplored Bolivian Amazon region and their potential for coffee diseases control

**DOI:** 10.1093/femsle/fnaf142

**Published:** 2025-12-22

**Authors:** Marisel M Mamani, Lilia Catacora, Nélida Nina, Wendy D Tola, Feng M Cai, Jesper Rydén, Irina S Druzhinina, Dan Funck Jensen, Carla F Crespo, Magnus Karlsson, Mukesh Dubey

**Affiliations:** Forest Mycology and Plant Pathology, Swedish University of Agricultural Sciences, 75007, Uppsala, Sweden; Área de Biotecnología Microbiana, Instituto de Investigaciones Fármaco Bioquímicas, Facultad de Ciencias Farmacéuticas y Bioquímicas, Universidad Mayor de San Andrés, 000, La Paz, Bolivia; Área de Biotecnología Microbiana, Instituto de Investigaciones Fármaco Bioquímicas, Facultad de Ciencias Farmacéuticas y Bioquímicas, Universidad Mayor de San Andrés, 000, La Paz, Bolivia; Área de Biotecnología Microbiana, Instituto de Investigaciones Fármaco Bioquímicas, Facultad de Ciencias Farmacéuticas y Bioquímicas, Universidad Mayor de San Andrés, 000, La Paz, Bolivia; Área de Biotecnología Microbiana, Instituto de Investigaciones Fármaco Bioquímicas, Facultad de Ciencias Farmacéuticas y Bioquímicas, Universidad Mayor de San Andrés, 000, La Paz, Bolivia; School of Ecology, Sun Yat-sen University, 518000, Shenzhen, China; Department of Energy and Technology, Swedish University of Agricultural Sciences, 75651, Uppsala, Sweden; Department Accelerated Taxonomy, Kew Science, Royal Botanic Gardens, Kew, TW9 3AB, Richmond, United Kingdom; Forest Mycology and Plant Pathology, Swedish University of Agricultural Sciences, 75007, Uppsala, Sweden; Área de Biotecnología Microbiana, Instituto de Investigaciones Fármaco Bioquímicas, Facultad de Ciencias Farmacéuticas y Bioquímicas, Universidad Mayor de San Andrés, 000, La Paz, Bolivia; Forest Mycology and Plant Pathology, Swedish University of Agricultural Sciences, 75007, Uppsala, Sweden; Forest Mycology and Plant Pathology, Swedish University of Agricultural Sciences, 75007, Uppsala, Sweden

**Keywords:** biofungicide, Caranavi-Yungas, coffea arabica, coffee rhizosphere, DNA barcode, disease management, fungal diversity, intraspecies variation

## Abstract

*Trichoderma* fungi are colonizers of plant substrates and rhizosphere and are valued for their antagonism against phytopathogens and ability to promote plant health. We investigated *Trichoderma* diversity in coffee-growing soils in Caranavi region of Yungas-La Paz, Bolivia, where high humidity and fungal diseases threaten yield, and evaluated their potential as biocontrol agents against coffee pathogens. A total of 440 *Trichoderma* were isolated from coffee rhizosphere, fallow lands, and forest ecosystems across an altitudinal gradient in Caranavi. DNA barcode analyses using ITS, *rpb2*, and *tef1* loci identified only four species. However, 47 taxa comprising 344 isolates were ambiguous, and 41 isolates were previously unrecognised species. The diversity of *Trichoderma* spp. was significantly affected by ecosystem type and altitude, with more species isolated from coffee rhizosphere than fallow lands and forest ecosystems, and from lower altitudes than higher ones. Evaluation of 100 isolates against a native coffee wilt pathogen *Fusarium oxysporum* identified 70 potent antagonists, with 30 achieving 90–100% disease control. This is the first comprehensive study of *Trichoderma* diversity in Yungas, identifying indigenous *Trichoderma* for biocontrol applications against coffee diseases. It also emphasizes the need to refine the *Trichoderma* species concept and improve the taxonomic resolution within the genus.

## Teaser

An investigation of *Trichoderma* diversity in the unexplored Amazonic region of Bolivia highlights the importance of refining the Trichoderma species concept and improving taxonomic resolution within the genus.

## Introduction

Fungal species from the genus *Trichoderma* (*Hypocreales, Sordariomycetes, Ascomycota*) are among the most isolated saprotrophic fungi across diverse ecosystems and play a role in ecological balance by decomposing organic matter and enhancing nutrient cycling (Druzhinina et al. 2011, Singh et al. 2020). Their wide distribution across various ecological niches, including soil, dead wood and decaying organic matter, and rhizosphere underscores their ability to thrive under different ecological conditions (Druzhinina et al. 2011, Singh et al. 2020). Many *Trichoderma* species thrive in rhizosphere and can antagonize and directly parasitize several plant-pathogenic fungi (Harman 2006, Druzhinina et al. 2011, Singh et al. 2020, Castillo et al. 2022). Additionally, they can establish beneficial relationships with plants by colonizing plant roots, promoting plant growth and inducing plant immune response (Harman 2006, Druzhinina et al. 2011, Singh et al. 2020, Castillo et al. 2022). Due to these characteristics, certain *Trichoderma* strains are frequently used to develop biocontrol products against a broad range of fungal pathogens (Harman 2006, Druzhinina et al. 2011, Singh et al. 2020, Castillo et al. 2022).

*Coffea arabica* (Arabica coffee) and *C. canephora* (Robusta coffee) are two economically significant species of the *Coffea* (Rubiaceae, Plantae) genus, with *C. arabica* accounting for the majority of global production in over 80 countries, including Bolivia (Somarriba et al. 2004). Coffee cultivation in Bolivia occurs mainly in the Caranavi province of the Yungas region, a mountainous area east of the mountain Andes and a transition zone between the highlands and the tropical lowlands of the Amazon basin. The high altitudes (range masl), unique climatic conditions, and fertile soil of the Yungas are ideal for growing high-quality Arabica coffee. In this region, coffee plants are mainly cultivated organically by smallholder farmers, excluding chemical fertilizers and fungicides. In recent years, fungal diseases have severely impacted coffee production in Caranavi. Common diseases in the region include coffee leaf spot (*Cercospora coffeicola; Capnodiales, Dothideomycetes, Ascomycota*), coffee rust (*Hemileia vastatrix; Pucciniales, Pucciniomycetes, Basidiomycota*), American leaf spot (*Mycena citricolor, Agaricales, Agaricomycetes, Basidiomycota*), leaf blight (*Corticium koleroga, Corticiales, Agaricomycetes, Basidiomycota*), coffee wilt (*Fusarium xylarioides, Hypocreales, Sordariomycetes*) and anthracnose (*Colletotrichum* spp.; *Glomerellales, Sordariomycetes*). These diseases often co-occur, creating complex management challenges for coffee growers, reducing yields, and compromising coffee quality (Yujra Serna 2016). Leveraging indigenous natural enemies, such as those belong to the genus *Trichoderma*, is viewed as a promising approach for sustainable management of coffee diseases.

Recent advances in molecular methods for fungal identification and species delimitation, such as genealogical concordance phylogenetic species recognition (Taylor et al. 2000) and DNA barcoding analysis, have significantly contributed to the taxonomy of *Trichoderma* genus, enabling more accurate species identification (Cai and Druzhinina 2021). Recently, Cai and Druzhinina proposed a protocol and guidelines for molecular identification of *Trichoderma* species involving the internal transcribed spacer (ITS), translation elongation factor 1 alpha (*tef1*), and RNA polymerase B subunit II (*rpb2*) loci as DNA barcodes (Cai and Druzhinina 2021). Currently, more than 500 *Trichoderma* species have been reported (www.speciesfungorum.org; Bissett et al. 2015, Cai and Druzhinina 2021). *Trichoderma* species have been isolated from the rhizosphere of many crop plants, including coffee (Mulaw et al. 2010, Rodríguez et al. 2021, Mulatu et al. 2023). Specific isolates of *T. asperellum* and *T. longibrachiatum* from the coffee rhizosphere have shown promising antagonistic and biocontrol potential against coffee pathogens (Assefa et al. 2021, Mulatu et al. 2023). However, the occurrence and distribution of *Trichoderma* species in coffee-growing regions of Bolivia, and their potential application for the biocontrol of coffee diseases, remain unknown.

This study aims to characterize the diversity of *Trichoderma* in the coffee-growing regions of Yungas in Bolivia, focusing on coffee rhizosphere, fallow land, and pristine forests. Additionally, we aim to assess the antagonistic potential of local *Trichoderma* isolates to manage coffee pathogens in Bolivia, thereby contributing to the development of sustainable and resilient coffee production systems. We hypothesize that i) both altitude and ecosystem type significantly influence the diversity of *Trichoderma* species, and ii) *Trichoderma* isolates will exhibit antagonistic abilities against coffee pathogens. In total, 440 *Trichoderma* isolates were collected, identified using ITS, *tef1*, and *rpb2* barcode loci. Diversity and abundance of *Trichoderma* species were positively correlated with soil organic content. Furthermore, an evaluation of 100 *Trichoderma* isolates demonstrated that 70 isolates exhibited significant *in vitro* antagonistic ability against a native coffee pathogen *Fusarium oxysporum*. This research assesses *Trichoderma* diversity in the Yungas region of Bolivia and highlights the potential application of indigenous *Trichoderma* isolates for the biocontrol of coffee fungal pathogens.

## Material and methods

### Study area and soil sampling

**Figure 1.**
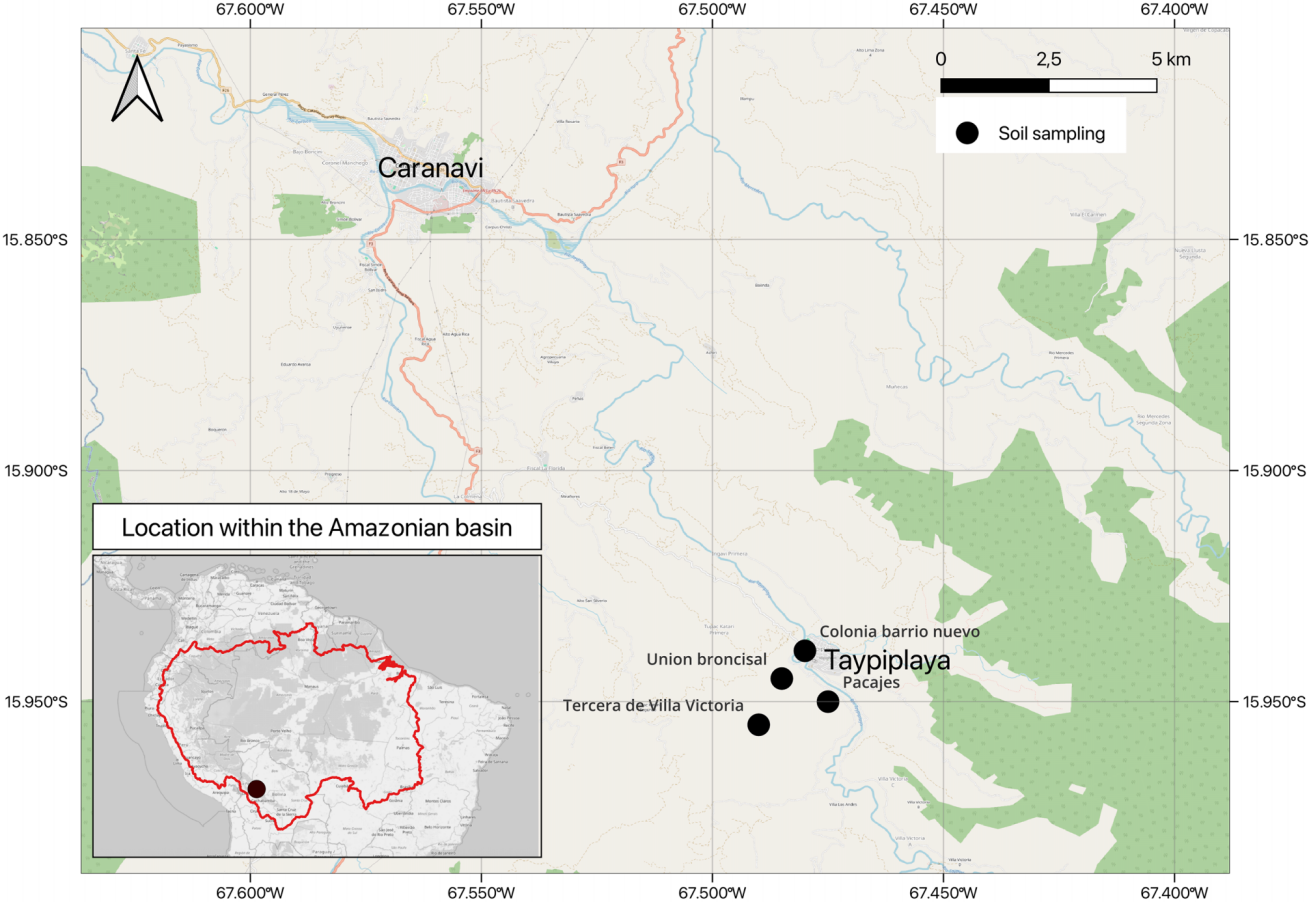
Geographic location of the sampling area in the Yungas region, Bolivia. Soil samples were collected in *Taipiplaya* district of Caranavi municipality (black point) from coffee rhizosphere, fallow land, and forest soils across three altitudinal zones (high, mid, and low). The map was created using Quantum Geographic Information System (QGIS). Data source: © OpenStreetMap contributors (2025) under the Open Database License (ODbL).

Ninety four soil samples were collected from coffee producing areas, fallow lands and pristine forests in four communities located at different altitudes: 1) Colonia Barrio Nuevo, 2) Unión Broncisal, both located in high altitude areas (1680 masl); 3) Tercera de Villa Victoria, located in a medium altitude area (1450 masl); and 4) Pacajes, located in a low altitude area (500 masl). These communities are located in Taipiplaya district in the municipality of Caranavi, La Paz Bolivia. Taipiplaya is located on the eastern edge of the Cordillera Real, in the Bolivian Yungas, a transition region between the Andean highlands and the Amazonian lowlands (Fig. 1). The area extends along a small valley east of Caranavi, surrounded by mountain ranges up to 2500 masl (Chuquimia Rojas and Arellano López 2013). The high-altitude areas encompass three distinct ecosystems: forested areas with protected green spaces, fallow land used for coffee cultivation, and coffee plantations. In the medium-altitude area, coffee plantations are adjacent to forested areas. In contrast, the low-altitude zone is dominated by agroforestry systems associated with coffee cultivation and fallow lands.

Soil samples from coffee plantations included the coffee rhizosphere and was collected from adult *C. arabica* plants of the Catuai variety grown at the Taipiplaya Coffee Growers Association (ASOCAFE) farm, which specializes in organic coffee production. Five hundred grams of soil samples were collected from the immediate area around coffee root systems, including the coffee roots, at a depth of approximately 50 cm, ensuring the inclusion of the first three soil horizons and the rhizosphere (Edwards et al. 2015). In the case of fallow and forest ecosystems, soil samples were collected after removing the surface layer of leaf litter. In total, 94 samples (40 from high altitude zone, 30 from medium altitude zone and 24 from low altitude zone) were collected. The soil samples were stored in sterile polyethene bags and kept at 4°C until further processing (Supplementary Table 1).

## Soil properties

Physicochemical characteristics of soil samples were analyzed in three replicates. Soil samples were ground and passed through a 2 mm sieve. Soil texture was determined by the Bouyoucos method (Beretta et al. 2014). Soil colour was determined using the Munsell soil colour charts (Munsell Color 2000). Soil pH and electrical conductivity were measured with a pH meter (Ohaus, Ohaus Corporation, USA) and a conductivity meter (Portable Meter Kit, Oakton Instruments, Singapore) in a 1:2.5 soil: water suspension. Total nitrogen content was quantified using the Kjeldahl method (FAO 2021b). Total organic carbon content was determined using the Walkley-Black method (Walkley and Black 1934, FAO 2020). Available phosphorus in soil samples was determined using the Olsen method (FAO 2021a, Olsen et al. 1954).

## Isolation and purification of Trichoderma isolates

Soil suspensions were prepared by serial dilution to isolate *Trichoderma*. Ten grams of soil were added to 100 mL of sterile distilled water and homogenized on a rotary shaker for 30 minutes at room temperature. The resulting suspension was serially diluted from 10^–1^ to 10^–6^. From each dilution, 100 µL aliquots were spread onto potato dextrose agar (PDA; Sigma-Aldrich, St. Louis, MO) or selective Rose Bengal agar plates (Vargas Gil et al. 2009). The Rose Bengal medium was prepared (in g/L): 0.2 MgSO_4_·7H_2_O, 0.9 K_2_HPO_4_, 1.0 NH_4_NO_3_, 0.15 KCl, 0.15 Rose Bengal, 3.0 glucose, and 20 g agar (Williams et al. 2003). The pH of the medium was adjusted to 5.5 ± 0.1 before autoclaving at 121°C for 15 minutes (Williams et al. 2003). The plates were incubated at 25 ± 1°C in complete darkness for 72 hours. All isolation steps were performed in triplicate, with each soil sample plated onto three independent PDA or Rose Bengal agar plates.

Fungal colonies were monitored daily, and colonies resembling *Trichoderma* were aseptically transferred to PDA (Sigma-Aldrich, St. Louis, MO) supplemented with 0.4 g/L chloramphenicol (Sigma-Aldrich, St. Louis, MO). To obtain axenic cultures, spore suspensions from PDA-grown colonies were subjected to serial dilutions and single-spore purified.

### Ecophysiological profiling of *Trichoderma*

Radial growth rate of each isolate was determined on PDA. Mycelial plugs (5 mm diameter) were excised from the actively growing edges of a 5-day-old colony and centrally inoculated onto fresh PDA plates. All plates were incubated at 25 ± 1°C in darkness for up to 7 days. Three independent replicates were performed per isolate. Colony diameter was measured daily in two perpendicular directions using a millimetre ruler, and the mean value was calculated for each replicate. The radial growth rate (mm day⁻¹) was estimated.

Morphological characteristics, including colony morphology, pigmentation, and sporulation patterns, were recorded during incubation. Microscopic structures, such as conidiophores, phialides, and conidia, were examined in fresh preparations mounted in a 3% KOH solution and observed under a light microscope (Olympus CX43, Tokyo, Japan). Morphological traits were identified following the guidelines established by Samuels et al. (2002).

### DNA extraction, PCR amplification and sequencing

Fungal mycelium was cultured in potato dextrose broth (PDB, Difco™, Sparks, MD) for three days. Approximately 100–200 mg mycelium was transferred to a 2 mL tube containing 3 mm glass beads, and DNA was extracted following a cetyltrimethylammonium bromide-based method (Nygren et al. 2008). DNA concentration and purity were measured using a NanoDrop ND-1000 spectrophotometer (Thermo Fisher Scientific, Waltham, MA).

Amplification of the ITS region (White et al. 1990), *tef1* (O’Donnell et al. 1998), and *rpb2* (Liu et al. 1999) loci was performed using primer pairs ITS4/ITS5, EF1/EF2, and fRPB-5f/fRPB-7cr, respectively, following the protocols described by Cai and Druzhinina (Cai and Druzhinina 2021). Due to poor performance of the fRPB-5f/fRPB-7cr primers in our lab conditions, a new set of primers were designed (Supplementary Table 2), based on *Trichoderma rpb2* sequences from MycoCosm (https://mycocosm.jgi.doe.gov/mycocosm/home). In total, seven forward and six reverse primers were designed, and pooled in equimolar concentration. PCR reactions were set up in a final volume of 50 µL containing 12.5 ng of template DNA, 5 µL of 10X buffer, 5 µL of dNTP mix (2 mM), 2 µL of each primer (10 µM), 0.6 µL of Taq DNA polymerase (5 U/µL), and molecular-grade water to adjust the final volume. The thermal cycling program consisted of an initial denaturation at 95°C for 3 minutes, followed by 32 cycles of denaturation at 95°C for 30 seconds, annealing at 56°C for 30 seconds, and extension at 72°C for 1 minute, with a final extension at 72°C for 10 minutes. PCR products were separated by electrophoresis on a 1% agarose gel, and the expected fragment sizes confirmed amplifications: ∼700 bp for the ITS region, ∼900 bp for *tef1*, and ∼1200 bp for *rpb2*. The PCR products were purified using the AMPure XP PCR Purification Kit (Beckman Coulter, Brea, CA) according to the manufacturer’s protocol. The purified PCR products were subjected to Sanger sequencing at Macrogen Europe (Maastricht, The Netherlands).

### Molecular identification

For molecular species identification, ITS, *tef1* and *rpb2* sequences were subjected to analysis following the protocol outlined by Cai and Druzhinina (2021). Sequence quality was assessed using SeqMan Pro 17 (DNASTAR, Inc., Madison, WI, USA). The ITS, *tef1*, and *rpb2* loci DNA sequences were uniformly trimmed using TrichoMARK (Cai and Druzhinina 2021). The processed sequences were aligned using Muscle 3.8.31 (Edgar 2004), integrated into AliView 1.23 (Larsson 2014). Pairwise sequence similarity was calculated using Clustal Omega (EMBL-EBI, Hinxton, UK) against curated reference datasets.

Sequence similarity percentages were determined by submitting multiple sequence alignment matrices of the ITS*, tef1*, and *rpb2* loci to Clustal Omega (https://www.ebi.ac.uk/Tools/msa/clustalo/) for pairwise similarity calculations. An isolate was identified as *Trichoderma* if the ITS sequence reached a similarity threshold of ≥ 76% with the reference sequences. For species-level identification, the *rpb2* and *tef1* barcode markers were required to meet sequence similarity thresholds of ≥ 99% and ≥ 97%, respectively (Cai and Druzhinina 2021).

Phylogenetic analyses were conducted using maximum likelihood methodology in IQ-TREE 1.6.12 (Nguyen et al. 2015), with statistical support calculated via 1000 bootstrap replicates. The nucleotide substitution model was determined using ModelFinder (Kalyaanamoorthy et al. 2017) under the Bayesian Information Criterion (BIC). Maximum parsimony analysis was also performed in PAUP 4.0b10* with 1000 bootstrap replicates. Phylogenetic trees were visualized using FigTree v1.4.2 and annotated in CorelDraw 2017 (Corel, Ottawa, Canada).

Species identification was considered unambiguous, precise, and accurate if conditions ITS ≥ 76%, *rbp2* ≥ 99% and *tef1* ≥ 97% were fulfilled. In cases where ITS ≥ 76%, but only one region (*tef1* or *rpb2*) met the similarity threshold, and the phylogenetic analysis revealed close relationships (≥ 80% bootstrap support) with specific species, the isolates were classified as either *T*. aff. [species] or *T*. cf. [species] as outlined by Cai and Druzhinina (2021). Additionally, isolates with ITS ≥ 76%, but both *tef1* and *rpb2* sequence similarity percentages below the established thresholds, were classified as *T*. sp. [strain], and represent potentially novel species.

### Evaluation of antagonistic potential against coffee pathogens

One hundred *Trichoderma* isolates were selected to evaluate their ability to suppress lesion development caused by the necrotrophic leaf pathogen tentatively identified as *Fusarium oxysporum* isolated from Coffee leaves, by the detached coffee leaf assay.

A spore suspension of *Trichoderma* isolates or pathogen (1 × 10^6^ conidia/mL) was prepared in sterile distilled water and adjusted using a hemocytometer (GmbH & Co. KG, Lauda-Königshofen, Germany). Coffee leaves (*C. arabica*, variety Catuai) were collected from coffee plants not older than 5 years. Leaves measured approximately 12–15 cm in length and were surface disinfected with 1% sodium hypochlorite (VWR, Radnor, PA) washed with sterilized water and air dried in a sterile cabinet for 24 hours before being dipped in the spore suspension of each *Trichoderma* isolate. After 24 hours, *Fusarium* spore suspension was sprayed onto the leaves pre-treated with *Trichoderma*. Leaves treated with sterile distilled water alone and the pathogen spore suspension were used as negative and positive controls, respectively. Treated leaves were incubated at 28 ± 1°C in sterile, moist chambers within plastic trays, enclosed in Ziploc bags to increase humidity. Each treatment consisted of five replicates (one leaf per replicate), and the experiment was repeated three times. Lesion development was assessed five days post-inoculation by measuring lesion size using the software CompuEye (Bakr 2005).

### Statistical methodology

Statistical analysis was performed using a one-way ANOVA on the data obtained from detached leaves assay to determine significant (*P* ≤ 0.05) treatment differences, followed by Tukey’s HSD test for pairwise comparisons. To investigate whether soil properties and ecosystems influence *Trichoderma* diversity and abundance, a generalised linear model (GLM) using the negative binomial family was applied, using implementation in R (Venables and Ripley 2002,R Core Team 2025). The responses are counts, and initial attempts with a Poisson distribution resulted in considerable overdispersion, which motivated the negative binomial distribution. Within this framework, backward elimination was used.

## Results

### Isolation and identification of *Trichoderma* species

In this study, 485 putative *Trichoderma* isolates were purified from 94 soil samples collected from the coffee (*C. arabica*) rhizosphere, forest soils, and fallow lands of the high, medium, and low altitude zones (Supplementary Table 3). Successful PCR amplification and sequencing of the ITS locus placed 440 isolates in the genus *Trichoderma* based on a sequence similarity ≥ 76% (Supplementary Table 4) when compared with the *Trichoderma* reference set from Cai and Druzhinina (2021). Among those, 331 were isolated from the coffee plant rhizosphere, including 127 isolates from the low zone, 71 from the medium zone and 133 from the high zone. The number of *Trichoderma* isolates from fallow land and forest soil was 80 and 29, respectively (Table 1).

**Table 1.** Distribution of *Trichoderma* isolates recovered from coffee rhizosphere, fallow land, and forest soils across low-, medium-, and high-altitude zones in Taipiplaya, Caranavi, Bolivia^#^.

Ecosystem	Low-altitude zone (500 masl)	Medium-altitude zone (1450 masl)	High-altitude zone (1580–1680 masl^§^)	Number of isolates
Rhizosphere coffee	127	71	133	331
Fallow soil	61	0	19	80
Forest soil	0	15	14	29
**Total** **^§^**	**188**	**86**	**166**	**440**

# The isolates were identified following the protocol suggested in Cai and Druzhinina (2021).

§ Values indicate the total number of isolates collected per altitudinal zone. masl: meters above sea level.

Out of the 440 isolates assigned to the genus *Trichoderma*, 330 isolates met the *rpb2* criterion (sequence similarity ≥ 99% compared with the *Trichoderma* reference set from Cai and Druzhinina 2021), and 198 of those also fulfilled the *tef1* criterion (sequence similarity ≥ 97%) (Table 2). This enabled the identification of four known *Trichoderma* species: *T. ghanense, T. pseudopyramidale, T. heveae*, and *T. rugulosum*, comprising 33 isolates (Table 3). However, 105 isolates with *rpb2* sequence similarity of ≥ 99% showed < 97% sequence similarity with *tef1*, while 30 isolates showed *rpb2* sequence similarity of < 99% but ≥ 97% *tef1* sequence similarity. This resulted in the identification of an additional 47 species, consisting of 22 *T*. cf. (104 isolates) and 25 *T*. aff. (241 isolates) (Table 3). Representative isolates were designated as *T*. aff. (*T*. aff. *brevicompactum, T*. aff. *brasiliensis, T*. aff. *pseudopyramidale, T*. aff. *ghanense, T*. aff. *orientale, T*. aff. *lentiforme, T*. aff. *afarasin, T*. aff. *jaklitschii, T*. aff. *rugulosum, T*. aff. *insigne, T*. aff. *simplex, T*. aff. *heveae, T*. aff. *koningiopsis, T*. aff. *acreanum, T*. aff. *peruvianum, T*. aff. *pinicola, T*. aff. *sparsum, T*. aff. *rifaii, T*. aff. *canadense, T*. aff. *endophyticum, T*. aff. *dorothopsis, T*. aff. *phayaoense, T*. aff. *ararianum, T*. aff. *zelobreve*) or *T*. cf. (*T*. cf. *brasiliensis, T*. cf. *pseudopyramidale, T*. cf. *orientale, T*. cf. *lentiforme, T*. cf. *afarasin, T*. cf. *jaklitschii, T*. cf. *subazureum, T*. cf. *insigne, T*. cf. *simplex, T*. cf. *heveae, T*. cf. *obovatum, T*. cf. *uncinatum, T*. cf. *koningiopsis, T*. cf. *acreanum, T*. cf. *peruvianum, T*. cf. *pinicola, T*. cf. *sparsum, T*. cf. *caribbaeum, T*. cf. *canadense, T*. cf. *nigricans, T*. cf. *hortense*) (Table 3). Additionally, 41 isolates did not meet the thresholds for *rpb2* and *tef1* and were designated *T*. sp. [strain] (Table 4). Phylogenetic analyses based on individual *rpb2* and *tef1* datasets consistently showed that these isolates formed 16 well-supported monophyletic clusters, each representing distinct phylogenetic lineages without close correspondence to any described *Trichoderma* species. These results strongly suggest that these isolates represent putative novel taxa within the genus *Trichoderma* (Supplementary Figure 1, Table 4).

**Table 2.** Distribution of *Trichoderma* isolates by taxonomic category across ecosystems and altitudinal zones in Taipiplaya, Bolivia.

Altitude	Ecosystem	ID^#^	*T*. cf*.^#^*	*T*. aff*.^#^*	*T. sp.^#^*	T. sp.*	No. of isolates
High (1580-1680 masl) zones	Rhizosphere Coffee	17	30	75	7	4	133
	Fallow soil	0	5	7	5	2	19
	Forest soil	3	5	4	2	0	14
Medium (1450 masl) zone	Rhizosphere Coffee	1	14	48	7	1	71
	Forest soil	1	3	7	3	1	15
Low (500 masl) zone	Rhizosphere Coffee	7	34	67	9	10	127
	Fallow soil	4	13	33	8	3	61
	**Total**	**33**	**104**	**241**	**41**	**21**	**440**

**# ID** indicates *Trichoderma* isolates successfully identified to species level; ***T*. cf**.: (*confer*) indicates molecular similarity but not full identity to known species; ***T*. aff**.: (*affinis*) indicates phylogenetic affinity but lacking full sequence identity; ***T. sp*.:** putative new species pending formal description; based on the *Trichoderma* DNA barcoding framework proposed by Druzhinina and Cai (2021). *T. sp*.*: Sequence ambiguous due to overlapping peaks or background noise. masl: meters above sea level.

**Table 3.** *Trichoderma* species identified by DNA barcoding and their distribution across altitudinal zones and ecosystems in Taipiplaya, Caranavi, Bolivia.

Species	Altitude^§^	Ecosystem	Species name	Number of isolates
**ID** **^#^**				
1	High	Rhizosphere Coffee	*T. ghanense*	3
	Low	Rhizosphere Coffee	*T. ghanense*	2
2	High	Rhizosphere Coffee	*T. pseudopyramidale*	14
		Soil forest	*T. pseudopyramidale*	3
	Medium	Rhizosphere Coffee	*T. pseudopyramidale*	1
		Soil forest	*T. pseudopyramidale*	1
	Low	Rhizosphere Coffee	*T. pseudopyramidale*	4
		Soil Fallow	*T. pseudopyramidale*	1
3	Low	Rhizosphere Coffee	*T. heveae*	1
4	Low	Soil Fallow	*T. rugulosum*	3
**Total**			**4 species**	**33**
***T*. cf**.				
5	Low	Rhizosphere Coffee	*T*. cf*. brasiliensis*	3
6	High	Rhizosphere Coffee	*T*. cf*. pseudopyramidale*	6
		Soil forest	*T*. cf*. pseudopyramidale*	3
	Medium	Rhizosphere Coffee	*T*. cf. *pseudopyramidale*	3
	Low	Rhizosphere Coffee	*T*. cf*. pseudopyramidale*	7
		Soil Fallow	*T*. cf*. pseudopyramidale*	1
7	High	Rhizosphere Coffee	*T*. cf*. ghanense*	2
	Medium	Soil forest	*T*. cf*. ghanense*	1
	Low	Rhizosphere Coffee	*T*. cf*. ghanense*	2
8	High	Rhizosphere Coffee	*T*. cf*. orientale*	4
		Soil forest	*T*. cf*. orientale*	2
		Soil Fallow	*T*. cf*. orientale*	4
	Medium	Rhizosphere Coffee	*T*. cf*. orientale*	1
	Low	Rhizosphere Coffee	*T*. cf*. orientale*	5
		Soil Fallow	*T*. cf*. orientale*	2
9	High	Rhizosphere Coffee	*T*. cf*. lentiforme*	1
	Low	Rhizosphere Coffee	*T*. cf*. lentiforme*	2
10	High	Rhizosphere Coffee	*T*. cf*. afarasin*	1
	Medium	Rhizosphere Coffee	*T*. cf*. afarasin*	1
11	High	Rhizosphere Coffee	*T*. cf*. jaklitschii*	3
	Medium	Rhizosphere Coffee	*T*. cf*. jaklitschii*	1
	Low	Rhizosphere Coffee	*T*. cf*. jaklitschii*	4
12	High	Rhizosphere Coffee	*T*. cf*. subazureum*	1
	Medium	Rhizosphere Coffee	*T*. cf*. subazureum*	3
	Low	Rhizosphere Coffee	*T*. cf*. subazureum*	1
		Soil Fallow	*T*. cf*. subazureum*	1
13	High	Rhizosphere Coffee	*T*. cf*. insigne*	4
	Medium	Rhizosphere Coffee	*T*. cf*. insigne*	1
	Low	Rhizosphere Coffee	*T*. cf*. insigne*	2
14	High	Rhizosphere Coffee	*T*. cf*. simplex*	1
	Medium	Soil forest	*T*. cf*. simplex*	1
15	Low	Rhizosphere Coffee	*T*. cf*. heveae*	5
16	High	Rhizosphere Coffee	*T*. cf*. obovatum*	1
17	High	Rhizosphere Coffee	*T*. cf*. uncinatum*	1
	Low	Soil Fallow	*T*. cf*. uncinatum*	1
18	Low	Rhizosphere Coffee	*T*. cf. *koningiopsis*	1
19	High	Rhizosphere Coffee	*T*. cf. *acreanum*	2
	Medium	Rhizosphere Coffee	*T*. cf*. acreanum*	2
20	High	Rhizosphere Coffee	*T*. cf*. peruvianum*	3
		Soil Fallow	*T*. cf*. peruvianum*	1
	Medium	Rhizosphere Coffee	*T*. cf*. peruvianum*	2
	Low	Rhizosphere Coffee	*T*. cf*. peruvianum*	1
		Soil Fallow	*T*. cf*. peruvianum*	2
21	Low	Rhizosphere Coffee	*T*. cf*. pinicola*	1
22	Low	Soil Fallow	*T*. cf*. sparsum*	1
23	Medium	Soil forest	*T*. cf*. caribbaeum*	1
24	Low	Soil Fallow	*T*. cf. *canadense*	1
25	Low	Soil Fallow	*T*. cf. *nigricans*	2
26	Low	Soil Fallow	*T*. cf*. hortense*	2
**Total**			**22 species**	**104**
***T*. aff**.				
27	Low	Rhizosphere Coffee	*T*. aff*. brevicompactum*	2
28	Low	Rhizosphere Coffee	*T*. aff*. brasiliensis*	3
29	High	Rhizosphere Coffee	*T*. aff*. pseudopyramidale*	6
		Soil Fallow	*T*. aff*. pseudopyramidale*	2
	Medium	Rhizosphere Coffee	*T*. aff*. pseudopyramidale*	1
	Low	Rhizosphere Coffee	*T*. aff*. pseudopyramidale*	1
30	High	Rhizosphere Coffee	*T*. aff*. ghanense*	9
		Soil forest	*T*. aff*. ghanense*	1
	Medium	Rhizosphere Coffee	*T*. aff*. ghanense*	3
		Soil forest	*T*. aff*. ghanense*	1
	Low	Rhizosphere Coffee	*T*. aff*. ghanense*	6
		Soil Fallow	*T*. aff*. ghanense*	2
31	High	Rhizosphere Coffee	*T*. aff. *orientale*	4
		Soil forest	*T*. aff*. orientale*	1
	Low	Rhizosphere Coffee	*T*. aff*. orientale*	4
		Soil Fallow	*T*. aff*. orientale*	3
32	High	Rhizosphere Coffee	*T*. aff*. lentiforme*	12
	Medium	Rhizosphere Coffee	*T*. aff*. lentiforme*	3
		Soil Forest	*T*. aff*. lentiforme*	1
	Low	Rhizosphere Coffee	*T*. aff*. lentiforme*	14
		Soil Fallow	*T*. aff*. lentiforme*	10
33	High	Rhizosphere Coffee	*T*. aff*. afarasin*	6
		Soil Fallow	*T*. aff*. afarasin*	3
	Medium	Rhizosphere Coffee	*T*. aff*. afarasin*	9
	Low	Rhizosphere Coffee	*T*. aff*. afarasin*	8
		Soil Fallow	*T*. aff*. afarasin*	4
34	High	Rhizosphere Coffee	*T*. aff*. jaklitschii*	4
		Soil Fallow	*T*. aff*. jaklitschii*	1
	Medium	Rhizosphere Coffee	*T*. aff*. jaklitschii*	4
	Low	Rhizosphere Coffee	*T*. aff*. jaklitschii*	12
		Soil Fallow	*T*. aff*. jaklitschii*	2
35	High	Rhizosphere Coffee	*T*. aff*. rugulosum*	5
36	High	Rhizosphere Coffee	*T*. aff*. subazureum*	1
		Soil Forest	*T*. aff*. subazureum*	1
	Medium	Rhizosphere Coffee	*T*. aff*. subazureum*	2
		Soil Forest	*T*. aff*. subazureum*	2
	Low	Rhizosphere Coffee	*T*. aff*. subazureum*	1
37	High	Rhizosphere Coffee	*T*. aff*. insigne*	2
	Medium	Rhizosphere Coffee	*T*. aff*. insigne*	1
38	High	Rhizosphere Coffee	*T*. aff*. simplex*	1
	Medium	Rhizosphere Coffee	*T*. aff. *simplex*	2
39	Low	Rhizosphere Coffee	*T*. aff. *heveae*	2
40	High	Rhizosphere Coffee	*T*. aff*. koningiopsis*	8
		Soil Fallow	*T*. aff*. koningiopsis*	1
		Soil forest	*T*. aff. *koningiopsis*	1
	Medium	Rhizosphere Coffee	*T*. aff*. koningiopsis*	9
	Low	Rhizosphere Coffee	*T*. aff*. koningiopsis*	1
		Soil Fallow	*T*. aff*. koningiopsis*	2
41	High	Rhizosphere Coffee	*T*. aff*. acreanum*	8
	Medium	Rhizosphere Coffee	*T*. aff*. acreanum*	1
	Low	Rhizosphere Coffee	*T*. aff*. acreanum*	3
		Soil Fallow	*T*. aff*. acreanum*	1
42	High	Rhizosphere Coffee	*T*. aff*. peruvianum*	6
	Medium	Rhizosphere Coffee	*T*. aff*. peruvianum*	2
		Soil forest	*T*. aff. *peruvianum*	1
	Low	Soil Fallow	*T*. aff*. peruvianum*	1
43	Low	Rhizosphere Coffee	*T*. aff*. pinicola*	3
		Soil Fallow	*T*. aff. *pinicola*	1
44	Low	Rhizosphere Coffee	*T*. aff*. sparsum*	1
		Soil Fallow	*T*. aff*. sparsum*	1
45	Low	Rhizosphere Coffee	*T*. aff*. rifaii*	1
46	High	Rhizosphere Coffee	*T*. aff*. endophyticum*	2
	Medium	Rhizosphere Coffee	*T*. aff*. endophyticum*	6
		Soil forest	*T*. aff. *endophyticum*	2
47	Low	Rhizosphere Coffee	*T*. aff*. canadense*	1
		Soil Fallow	*T*. aff*. canadense*	4
48	Medium	Rhizosphere Coffee	*T*. aff. *dorothopsis*	4
	Low	Rhizosphere Coffee	*T*. aff*. dorothopsis*	3
		Soil Fallow	*T*. aff*. dorothopsis*	2
49	Medium	Rhizosphere Coffee	*T*. aff*. phayaoense*	1
50	High	Rhizosphere Coffee	*T*. aff. *ararianum*	1
51	Low	Rhizosphere Coffee	*T*. aff*. zelobreve*	1
**Total**			**25 species**	**241**

# **ID** indicates *Trichoderma* isolates successfully identified to species level; ***T*. cf**.: (*confer*) indicates molecular similarity but not full identity to known species; ***T*. aff**.: (*affinis*) indicates phylogenetic affinity but lacking full sequence identity; based on the *Trichoderma* DNA barcoding framework proposed by Druzhinina and Cai (2021).

§ Altitudinal zones correspond to low (≈ 500 masl), medium (≈ 1450 masl), and high (1580–1680 masl).

**Table 4.** The list of *Trichoderma* isolates failed to meet the thresholds for *rpb2* and *tef1* and were designated as putative new species *T*. sp^#^.

			Similarity standard—ITS			Similarity standard—*rpb2*			Similarity standard—*tef1*			
No	Strain ID	Ecosystem^§^	Genus ITS *≥ 76*	Similarity	Max (Score)	Species *rpb2 ≥ 99*	Similarity	Max (Score)	Species *tef1 ≥ 97*	Similarity	Max (Score)	Identified
1	BT 105–2	RC-L	*Trichoderma*	*T. intricatum*	81.55	***−***	*T. dorothopsis*	98.77	***−***	*T. arenarium*	95.72	*T*. sp.
2	AT 51	Sfo-H	*Trichoderma*	*T. ovalisporum*	99.22	***−***	*T orientale*	98.89	***−***	*T. austriacum*	35.17	*T*. sp.
3	AT 6–3	RC-H	*Trichoderma*	*T. linzhiense*	99.43	***−***	*T. longibrachiatum f sp bissettii*	98.83	***−***	*T. orientale*	95.33	*T*. sp.
4	AT 14–1	Sfa-H	*Trichoderma*	*T. longibrachiatum*	98.72	***−***	*T. pyramidale*	98.15	***−***	*T. orientale*	87.26	*T*. sp.
5	ST 33	RC-H	*Trichoderma*	*T. gamsii*	100	***−***	*T. orientale*	98.77	***−***	*T. arenarium*	96.08	*T*. sp.
				*T. koningiopsis*								
				*T. ovalisporum*								
6	BT 72	Sfa-L	*Trichoderma*	*T. longibrachiatum*	93.62	***−***	*T. longibrachiatum f sp bissettii*	98.83	***−***	*T. orientale*	86.84	*T*. sp.
7	MT 48	RC-M	*Trichoderma*	*T. longibrachiatum*	98.55	***−***	*T. longibrachiatum f sp bissettii*	98.83	***−***	*T. orientale*	95.32	*T*. sp.
8	MT 12–2	Sfo-M	*Trichoderma*	*T. virens*	93.28	***−***	*T. ararianum*	98.45	***−***	*T. inaequilaterale*	96.99	*T*. sp.
9	AT 26–1	RC-H	*Trichoderma*	*T. longibrachiatum*	98.36	***−***	*T. acreanum*	98.96	***−***	*T. orientale*	95.32	*T*. sp.
10	AT 74	RC-H	*Trichoderma*	*T. longibrachiatum*	96.36	***−***	*T. longibrachiatum f sp bissettii*	98.64	***−***	*T. orientale*	95.54	*T*. sp.
11	HT 27–2	RC-M	*Trichoderma*	*T. lentiforme*	99.81	***−***	*T. pseudopyramidale*	98.99	***−***	*T. pseudopyramidale*	91.8	*T*. sp.
12	LT 17	RC-L	*Trichoderma*	*T. lentiforme*	100	***−***	*T. longibrachiatum f sp bissettii*	98.83	***−***	*T. lentiforme*	94.35	*T*. sp.
13	AT 19–2	RC-H	*Trichoderma*	*T. gamsii*	100	***−***	*T. brunneoviride*	90.19	***−***	*T. acreanum*	96.66	*T*. sp.
				*T. koningiopsis*								
				*T. ovalisporum*								
14	BT 57–2	Sfa-L	*Trichoderma*	*T. lentiforme*	100	***−***	*T. longibrachiatum f sp bissettii*	98.83	***−***	*T. lentiforme*	83.33	*T*. sp.
15	MT 22	Sfo-M	*Trichoderma*	*T. virens*	93.28	***−***	*T. peltatum*	78.48	***−***	*T. inaequilaterale*	96.99	*T*. sp.
16	MT 1	RC-M	*Trichoderma*	*T. virens*	93.28	***−***	*T. densissimum*	96.68	***−***	*T. inaequilaterale*	96.99	*T*. sp.
17	LT 48–1	RC-L	*Trichoderma*	*T. guizhouense*	98.49	***−***	*T. longibrachiatum f sp bissettii*	98.64	***−***	*T. lentiforme*	96.14	*T*. sp.
18	BT 80–2	RC-L	*Trichoderma*	*T. longibrachiatum*	98.18	***−***	*T. awajun*	92.95	***−***	*T. orientale*	96.39	*T*. sp.
19	ST 20–2	RC-H	*Trichoderma*	*T. lentiforme*	97.92	***−***	*T. pseudopyramidale*	98.99	***−***	*T. pyramidale*	94.99	*T*. sp.
20	MT 7	Sfo-M	*Trichoderma*	*T. longibrachiatum*	97.45	***−***	*T. dorothopsis*	96.22	***−***	*T. pinnatum*	87.43	*T*. sp.
21	BT 111	Sfa-L	*Trichoderma*	*T. lentiforme*	100	***−***	*T. ararianum*	98.96	***−***	*T. hortense*	80.47	*T*. sp.
22	BT 8–2	Sfa-L	*Trichoderma*	*T. lentiforme*	99.24	***−***	*T. afarasin*	95.82	***−***	*T. lentiforme*	96.44	*T*. sp.
23	BT 5–2	Sfa-L	*Trichoderma*	*T. longibrachiatum*	98.54	***−***	*T. orientale*	98.77	***−***	*T. xanthum*	85.78	*T*. sp.
24	BT 84–2	Sfa-L	*Trichoderma*	*T. lentiforme*	99.62	***−***	*T. dorothopsis*	97.66	***−***	*T pseudostramineum*	52.04	*T*. sp.
25	AT 86	RC-H	*Trichoderma*	*T. longibrachiatum*	96.72	***−***	*T. peruvianum*	97.05	***−***	*T. orientale*	87.69	*T*. sp.
26	LT 30	RC-L	*Trichoderma*	*T. linzhiense*	99.43	***−***	*T. polyalthiae*	41.53	***−***	*T. zelobreve*	95.88	*T*. sp.
				*T. lentiforme*								
27	AT 14–2	Sfa-H	*Trichoderma*	*T. reeseiQM6a*	93.52	***−***	*T. andinense*	97.72	***−***	*T. euskadiense*	82.97	*T*. sp.
28	AT 64	Sfo-H	*Trichoderma*	*T. lentiforme*	99.79	***−***	*T. pseudopyramidale*	96.6	***−***	*T. pseudopyramidale*	89.78	*T*. sp.
29	AT 29–2	Sfa-H	*Trichoderma*	*T. longibrachiatum*	98.36	***−***	*T. orientale*	98.52	***−***	*T. orientale*	85.96	*T*. sp.
30	BT 101	RC-L	*Trichoderma*	*T. lentiforme*	100	***−***	*T. jaklitschii*	97.32	***−***	*T. lentiforme*	96.44	*T*. sp.
31	BT 38	Sfa-L	*Trichoderma*	*T. lentiforme*	100	***−***	*T. peruvianum*	97.05	***−***	*T. lentiforme*	96.74	*T*. sp.
32	BT 39–1	RC-L	*Trichoderma*	*T. lentiforme*	100	***−***	*WGST11*	95.45	***−***	*T. lentiforme*	96.14	*T*. sp.
33	AT 84–2	Sfa-H	*Trichoderma*	*T. longibrachiatum*	98.54	***−***	*T. orientale*	98.77	***−***	*T. orientale*	87.23	*T*. sp.
34	MT 10	RC-M	*Trichoderma*	*T. citrinoviride*	92.63	***−***	*T. andinense*	98.17	***−***	*T. andinense*	80.31	*T*. sp.
35	MT 30	RC-M	*Trichoderma*	*T. koningiopsis*	99.42	***−***	*T. flavipes*	43.27	***−***	*T. acreanum*	96.17	*T*. sp.
36	BT 12	RC-L	*Trichoderma*	*T. longibrachiatum*	98.72	***−***	*T. orientale*	98.89	***−***	*T. orientale*	86.2	*T*. sp.
37	MT 35–2	RC-M	*Trichoderma*	*T. longibrachiatum*	98.54	***−***	*T. orientale*	98.52	***−***	*T. orientale*	92.13	*T*. sp.
38	BT 42	Sfa-L	*Trichoderma*	*T. lentiforme*	100	***−***	*T. zelobreve*	95.08	***−***	*T. neocrassum*	68.41	*T*. sp.
39	AT 67	Sfa-H	*Trichoderma*	*T. longibrachiatum*	98.54	***−***	*T. orientale*	98.4	***−***	*T. pinnatum*	85.43	*T*. sp.
40	MT 35–1	RC-M	*Trichoderma*	*T. longibrachiatum*	98.54	***−***	*T. orientale*	98.52	***−***	*T. orientale*	95.53	*T*. sp.
41	BT 92	RC-L	*Trichoderma*	*T. longibrachiatum*	98.55	***−***	*T. orientale*	98.52	***−***	*T. orientale*	96.6	*T*. sp.

# The table lists *Trichoderma* isolates that did not meet the species-level similarity thresholds (*rpb2* ≥ 99%, *tef1* ≥ 97%) suggested in Cai and Druzhinina (2021). These isolates were therefore classified as *Trichoderma* sp. (*T*. sp.)and represent putative novel species pending further phylogenetic and morphological characterization.

§ RC-H = Rhizosphere coffee. high altitude; RC-M = Rhizosphere coffee. medium altitude; RC-L = Rhizosphere coffee. low altitude; SFo-H = Soil forest. high altitude; SFo-M = Soil Forest. medium altitude; SFa-H = Soil fallow land high altitude; SFa-L = Soil fallow low altitude

### *Trichoderma* diversity and abundance are influenced by soil properties ecosystems and altitudes

**Figure 2.**
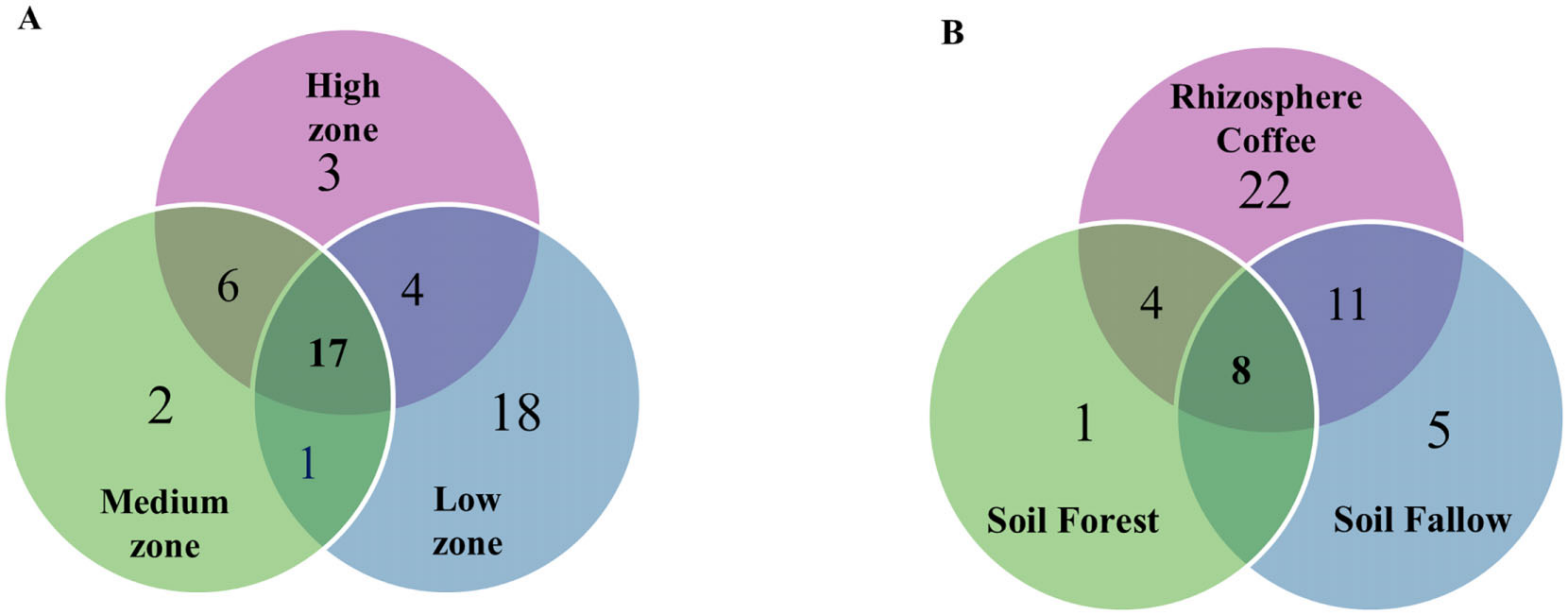
Diversity and distribution of *Trichoderma* species across altitudinal zones and soil ecosystems in Taipiplaya, Caranavi, Bolivia. (A) Distribution of *Trichoderma* species among high-, medium-, and low-altitude zones. (B) Distribution of *Trichoderma* species among rhizosphere coffee, fallow, and forest soils. Numbers indicate the total number of *Trichoderma* species occurring in each category, with overlaps showing species shared between zones or ecosystems.

The distribution analysis of *Trichoderma* species across the three altitude zones revealed distinct patterns of species occurrence. A total of 18 species were found exclusively in the low-altitude zone (*T. heveae, T. rugulosum, T*. cf*. brasiliensis, T*. cf*. heveae, T*. cf*. koningiopsis, T*. cf. *pinicola, T*. cf*. sparsum, T*. cf*. canadense, T*. cf*. nigricans, T*. cf*. hortense, T*. aff*. brevicompactum, T*. aff. *brasiliensis, T*. aff*. heveae, T*. aff*. pinicola, T*. aff*. sparsum, T*. aff*. rifaii, T*. aff*. canadense*, and *T*. aff*. zelobreve*). Only two species, *T*. cf*. caribbaeum* and *T*. aff*. phayaoense*, were exclusive to the medium-altitude zone, while three species (*T*. cf. *obovatum, T*. aff*. rugulosum*, and *T*. aff*. ararianum*) were restricted to the high-altitude zone (Fig. 2A, Supplementary Table 5).

Species shared between altitudinal zones were also detected, indicating a broader ecological amplitude for certain taxa. Six species were shared between the medium and high zones, one species occurred in both the medium and low zones, and four species were present in both the low and high zones. Notably, 17 species were widely distributed across all three altitude zones, highlighting their broad ecological adaptability and potential niche overlap (Fig. 2A, Supplementary Table 5).

Similarly, the distribution of *Trichoderma* species across the three ecosystems was analyzed. The rhizosphere of coffee plants harboured the highest species richness across all altitudinal zones, with most species associated with this niche. A total of 22 species were found exclusively in the coffee rhizosphere, including *T. heveae, T. rugulosum, T*. cf. *brasiliensis, T*. cf. *heveae, T*. cf*. koningiopsis, T*. cf. *pinicola, T*. cf. *sparsum, T*. cf*. canadense, T*. cf. *nigricans, T*. cf. *hortense, T*. aff*. brevicompactum, T*. aff*. brasiliensis, T*. aff*. heveae, T*. aff. *pinicola, T*. aff*. sparsum, T*. aff*. rifaii, T*. aff. *canadense, T*. aff. *zelobreve, T*. cf. *lentiforme, T*. aff. *insigne*, T. aff*. simplex*, and *T*. aff*. ararianum* (Fig. 2B, Supplementary Table 6). In contrast, five species (*T*. cf. *nigricans, T*. cf. *rugulosum, T*. cf. *hortense, T*. aff. *rifaii*, and *T*. aff. *dorothopsis*) were restricted to fallow land, and a single species, *T*. cf. *caribbaeum*, was found exclusively in forest soil (Fig. 2B, Supplementary Table 6).

Patterns of species sharing between ecosystems revealed 11 species present in both rhizosphere and fallow soils, and four species shared between rhizosphere and forest soils. Notably, eight species (*T. pseudopyramidale, T*. cf*. orientale, T*. aff*. orientale, T*. aff*. ghanense, T*. aff*. endophyticum, T*. cf*. peruvianum, T*. aff*. lentiforme*, and *T*. cf*. subazureum*) were consistently detected across all three ecosystems, highlighting their broad ecological tolerance and capacity to colonize diverse soil environments (Fig. 2B, Supplementary Table 6). These results demonstrate that the rhizosphere represents a hotspot of *Trichoderma* diversity in coffee-associated soils, while fallow and forest soils harbour a smaller, more specialized subset of the community.

To test whether soil characteristics and ecosystems influenced *Trichoderma* distribution, soil texture, organic carbon, pH, total nitrogen, phosphorus, and conductivity were analyzed across all soil samples (Supplementary Table 7). A GLM with the negative binomial family and backward elimination revealed significant effects of ecosystem type (*P* < 0.001), soil texture (*P* ≤ 0.006), and organic carbon (*P* ≤ 0.034) on *Trichoderma* diversity and abundance.

Species richness was consistently higher in coffee rhizosphere soils compared to fallow land and forest soils, confirming the importance of plant-associated niches. Moreover, soils with higher organic carbon (> 3.5%) supported a greater number of species (up to seven per sample), whereas soils with low carbon content (< 2.0%) harboured only one or two species. Similarly, *Trichoderma* abundance was higher in loamy and sandy loam soils, while clay-rich soils showed reduced richness and fewer isolates. Soil pH also played a role, with moderately acidic soils (pH 6.0–6.5) favouring diversity, while more acidic conditions (pH 3.9–4.2) were associated with lower values. These findings indicate that both ecosystem type and soil fertility parameters strongly shape *Trichoderma* communities, with organic matter availability and soil structure serving as key drivers of diversity.

### Antagonistic activity of *Trichoderma* isolates

Based on differences in morphological characteristics and growth rate, 100 *Trichoderma* isolates were selected for testing their biocontrol potential against a local necrotrophic pathogen isolated from coffee leaf, using a detached coffee leaf assay. The selected *Trichoderma* isolates comprised 76 from the coffee rhizosphere, 21 from forest soils, and three from fallow lands. These isolates represented 30 species, including 11 isolates of *T*. aff*. afarasin*, seven isolates of *T*. aff*. lentiforme*, seven *T. pseudopyramidale*, six *T*. cf*. orientale*, five *T*. cf*. acreanum* (Supplementary Table 8). The pathogen-inoculated leaves showed severe necrosis, with lesion areas reaching up to 100% of the leaf area, indicating successful infection in the detached leaf infection assay and the high virulence of the pathogen strain.

**Figure 3.**
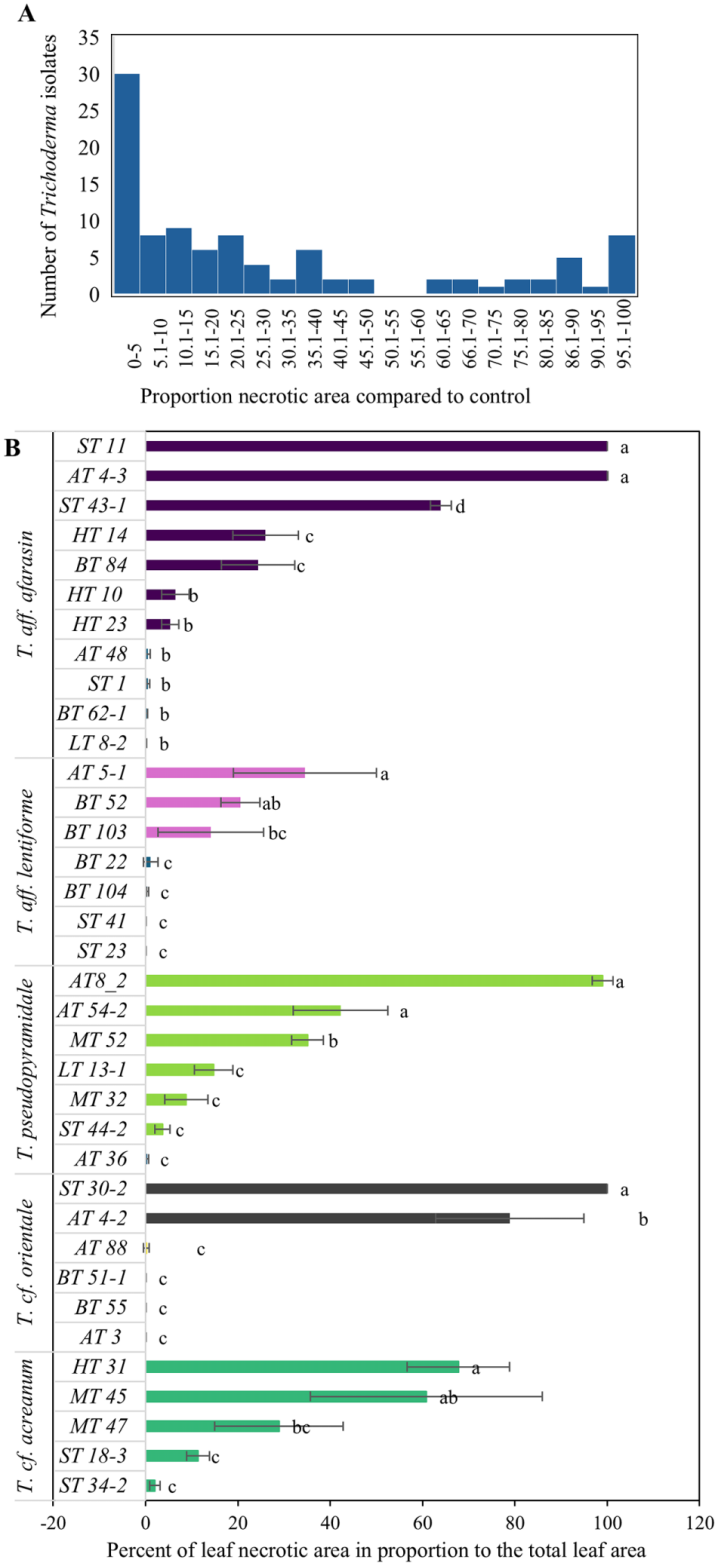
Intraspecific variation in the antagonistic activity of *Trichoderma* isolates against a native necrotrophic coffee pathogen *F. oxysporum* under detached leaf assay conditions. (A) Distribution of *Trichoderma* isolates according to the proportion of necrotic leaf area compared with the pathogen control (*F. oxysporum*). (B) Intraspecies variation in biocontrol efficacy among *Trichoderma* isolates. Bars represent mean ± SD (n = 5). Different letters above the bars indicate statistically significant differences according to Tukey’s HSD test (*P* < 0.05).

When applied to the leaves, 70 *Trichoderma* isolates significantly (*P* < 0.05) reduced coffee leaf lesion areas to varying degrees, demonstrating their potential as biocontrol agents against the pathogen (Fig. 3A, Supplementary Table 8). Among these, 30 isolates exhibited the highest antagonistic activity, reducing lesion areas by 90–100% (Fig. 3A). There was no correlation between the origin of *Trichoderma* isolates and their biocontrol ability. Interestingly, there was intraspecific variation in the biocontrol ability of *Trichoderma* isolates from several species under the tested conditions. For instance, four (AT 48, ST 1, BT 62–1, LT 8–2) out of 11 tested *T*. aff*. afarasin* isolates reduced lesion area by 95%, while ST 11 and AT 4–3 treated leaves showed no significant difference compared to the control (Fig. 3B). Similarly, four (BT 22, BT 104, ST 41, ST 23) out of seven tested *T*. aff. *lentiforme* isolates reduce the necrotic areas by more than 95%, while AT5-1 and BT 52 showed no effect (Fig. 3B). Application of four *T. pseudopyramidale* isolates (AT 36, ST 44–2, MT 32, LT 13–1) resulted in 85–100% reduced necrotic lesion area. At the same time, *T. pseudopyramidale* isolates AT 8–2, AT 54–2 and MT 52 showed no significant reduction compared to the control in the detached leaf assay. A similar intraspecific variation was obtained for *T*. cf*. orientale* and *T*. cf. *acreanum* isolates against the coffee pathogen under tested conditions (Fig. 3B).

## Discussion

In this study, we examined the diversity of *Trichoderma* in coffee-growing areas of the Caranavi province in the Yungas region of Bolivia. For identification, we applied the molecular barcode protocol outlined by Cai and Druzhinina (2021), where ITS is used as the primary barcode for genus verification followed by *tef1* and *rpb2* as secondary barcode loci for species identification. However, only four species were unambiguously, precisely, and accurately identified when applying this methodology. In contrast, the identification was ambiguous for 47 species. This relatively high proportion of ambiguous identification emphasize the importance of refining the *Trichoderma* species concept to advance the taxonomic precision within this genus. Caranavi belongs to Yungas province in La Paz, Bolivia, a transition area between the highlands and the tropical lowlands of the Amazon basin and has been virtually unexplored for fungal diversity. It may be assumed that this area contains *Trichoderma* genetic diversity previously unexplored by the scientific community, which can now be used to improve the accuracy of the *Trichoderma* molecular identification.

Genealogical concordance of several unlinked loci may help to assign unambiguous species names to some of the ambiguously identified species. The high genetic diversity of *Trichoderma* in the Yungas region is further emphasized by the 41 isolates that potentially represent new species. Here, the *Trichoderma* Whole Genus Genomic Project (TrichoCOSM), part of the US Department of Energy Joint Genome Institute’s Community Sequencing Program, where all described *Trichoderma* species will be genome-sequenced will further improve our understanding of species delineation and identification.

*Trichoderma* species are found globally across various ecosystems and climatic zones (Druzhinina et al. 2011, Kredics et al. 2014). Their occurrence is influenced by ecological factors, microclimate, substrate availability, and environmental interactions (Druzhinina et al. 2011, Kredics et al. 2014). Although *Trichoderma* species are cosmopolitan, their diversity and abundance are reported to be higher in the rhizosphere compared with non-rhizosphere soil (Druzhinina et al. 2011, Kredics et al. 2014). This is potentially due to their competitive ability, the presence of fungal hosts, and the availability of plant-derived nutrients, including root exudates. Numerous *Trichoderma* species have been isolated from the rhizosphere of various plants across diverse climatic zones, and many studies have reported *Trichoderma* diversity across various ecosystems (Kredics et al. 2014).

The occurrence and diversity of *Trichoderma* species in the coffee rhizosphere have also been reported, primarily from coffee-growing regions in Ethiopia (Mulaw et al. 2010, Rodríguez et al. 2021, Mulatu et al. 2022, de Sousa et al. 2022, Gomes et al. 2024). In addition to soil and rhizosphere, *Trichoderma* species have also been isolated from coffee leaves, berries, stems, and root systems (Vaga et al. 2010, Rodríguez et al. 2021). Our study evidenced a higher diversity of *Trichoderma* species, with 51 known species identified from 440 *Trichoderma* isolates collected from 94 samples, compared to previous reports from coffee rhizosphere in Ethiopia (Mulaw et al. 2010, Mulaw et al. (2010), Mulatu et al. 2022). identified eight known and eight putatively new species from 134 isolates from the coffee roots collected from different sites of four major coffee-growing agroecological systems in Ethiopia. These included *T. harzianum, T. hamatum, T. asperelloides, T. spirale, T. atroviride, T. koningiopsis, T. gamsii* and *T. longibrachiatum* (Mulaw et al. 2010). Additionally, Mulatu et al. (2022) identified 16 known *Trichoderma* species from 175 *Trichoderma* isolates from 184 coffee rhizosphere samples collected from 10 major Ethiopian coffee-growing areas, with a high prevalence of *T. harzianum, T. asperelloides* and T*. koningiopsis*. Intriguingly, the current study found no species reported from Ethiopia except *T. koningiopsis, T*. cf. *brevicompactum*, and *T*. cf*. orientale*. The high abundance of *T*. aff. *lentiforme, T. pseudopyramidale, T*. aff. *koningiopsis, T*. aff. *orientale, T*. aff. *ghanense* and *T*. cf. *orientale* found in this study differ from those previously reported from Ethiopia (Mulaw et al. 2010, Mulatu et al. 2022). This shows that the species diversity and richness of *Trichoderma* in the coffee-growing areas of the Yungas region in Bolivia differ from those in Ethiopia.

The differences in diversity and abundance of *Trichoderma* species are plausibly related to natural history and variations in soil characteristics and agricultural practices unique to each region, specifically between the Yungas regions in Bolivia and the coffee-growing areas of Ethiopia. For example, the soil samples collected in this study contained more organic carbon (3.0%–6.6%) and nitrogen (0.3%–7.6%) compared to the 1.6%–2.7% carbon and 0.19%–0.6% nitrogen reported in Ethiopian soil. Similarly, Bolivian soil ranges from sandy loam to clay sandy loam, whereas Ethiopian soil comprises sandy loam and silt loam (Mulaw et al. 2010). The importance of these soil characteristics is emphasized by the significant influence of soil organic carbon and texture on *Trichoderma* diversity and abundance in Yungas, which contrasts with a study from Ethiopia (Mulaw et al. 2010). High abundance of *T. koningiopsis* in samples collected from organically rich Colombian lowland Amazonian rainforest (Lopez-Quintero et al. 2013) is in line with our findings from Bolivia.

In Bolivia, altitude strongly influenced the distribution of *Trichoderma* species with a higher number of species identified in low-altitude areas compared to high-altitude zones, suggesting specific adaptations to environmental conditions such as temperature and organic matter availability. This is consistent with earlier reports from China, where the diversity of *Trichoderma* spp. was decreased with increasing altitude, and only a few strains were isolated from elevations above 3000 masl (Ma et al. 2020).

*Trichoderma* species are recognised for their biocontrol potential against various plant pathogens (Druzhinina et al. 2011). Similarly, certain *Trichoderma* isolates from the coffee rhizosphere have demonstrated a significant biocontrol effect against coffee wilt disease caused by *Fusarium xylarioides* (Mulatu et al. 2022). Additionally, these strains were reported to show high antagonism towards other phytopathogenic fungi, including the leaf pathogens *Alternaria alternata, Botrytis cinerea*, and *Sclerotinia sclerotiorum* (Mulaw et al. 2010, Mulaw et al. 2013, Mulatu et al. 2022). Our findings from the detached leaf assay suggest a biocontrol potential for the majority of the tested *Trichoderma* isolates, which aligns with previous results (Mulaw et al. 2010, Mulaw et al. 2013, Mulatu et al. 2022). The detached leaf assay data revealed high intraspecific variation in antagonistic ability among *Trichoderma* isolates, which has been reported previously for *T. asperellum* against coffee wilt pathogen *F. xylarioides* (Mulaw et al. 2013, Mulatu et al. 2022) and for *in vitro* nematocidal ability among isolates of the biocontrol fungus *Clonostachys rosea* (Iqbal et al. 2020). This suggests that factors contributing to biocontrol potential differ in a strain-specific manner in *Trichoderma*. This emphasizes the importance of evaluating intraspecific diversity in screening programmes when identifying new indigenous isolates for biocontrol applications. It also highlights the need for future research on the genetic basis for variation related to biocontrol traits, which will aid in identifying effective biocontrol agents against plant diseases.

## Conclusion

This study assesses the diversity and abundance of *Trichoderma* species across different ecosystems and altitudinal zones in coffee-growing communities in Caranavi province, Yungas region, Bolivia. Molecular analyses identified 51 species, indicating that the Yungas region harbours a high diversity of *Trichoderma* species. Species distribution varied by ecosystem and altitude, with rhizosphere soils supporting the highest *Trichoderma* diversity. Soil properties such as texture and organic carbon content significantly contributed to species diversity. A high proportion of the tested *Trichoderma* species showed an antagonistic potential suitable for biocontrol application against coffee pathogens. Intraspecies variation in the biocontrol ability of several *Trichoderma* species paves the way for future studies focusing on population genomics to understand the underlying mechanisms of biocontrol. Combining the ITS, *rpb2*, and *tef1* barcode loci with phylogenetic analysis offers a suitable method for identifying *Trichoderma* species. However, it needs further improvement, as some isolates did not fit within the existing classification criteria. It also highlights the importance of refining the *Trichoderma* species concept and enhancing the taxonomic resolution within the genus.

## Supplementary Material

fnaf142_Supplemental_Files

## Data Availability

The ITS sequence reported in this paper has been deposited in the National Centre for Biotechnology Information (NCBI) GenBank with accession no. SUB15727173. The *rpb2* and *tef1* sequences are submitted to the European Nucleotide Archive (ENA) under accession PRJEB101743.
