# Supplementary figures and images for: Diversity of Trichoderma in the unexplored Bolivian Amazon region and their potential for coffee diseases control
Source: FEMS Microbiol Lett. 2025 Dec 22;373:fnaf142. doi: 10.1093/femsle/fnaf142 (PMC12776340; doi:10.1093/femsle/fnaf142)

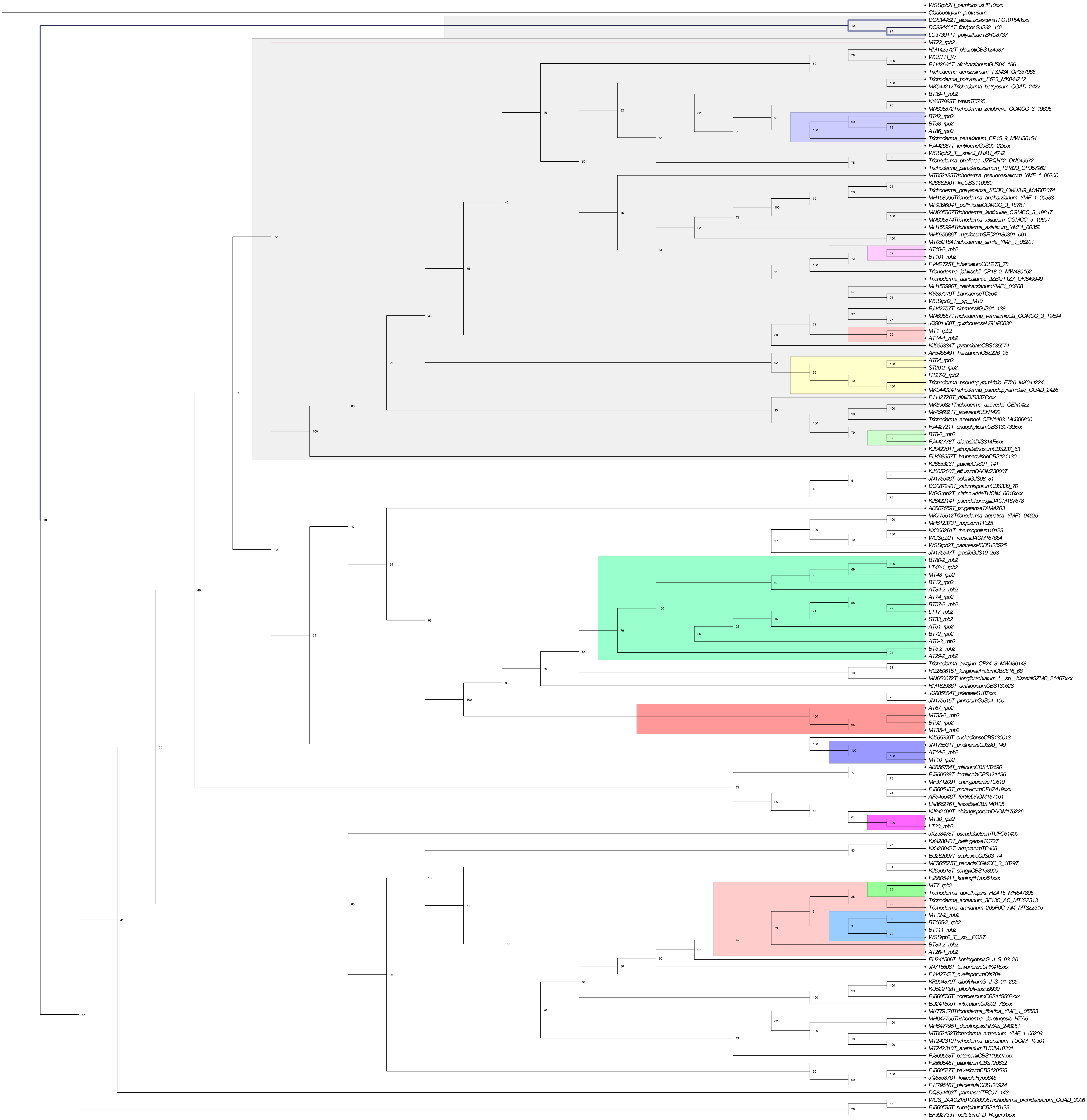

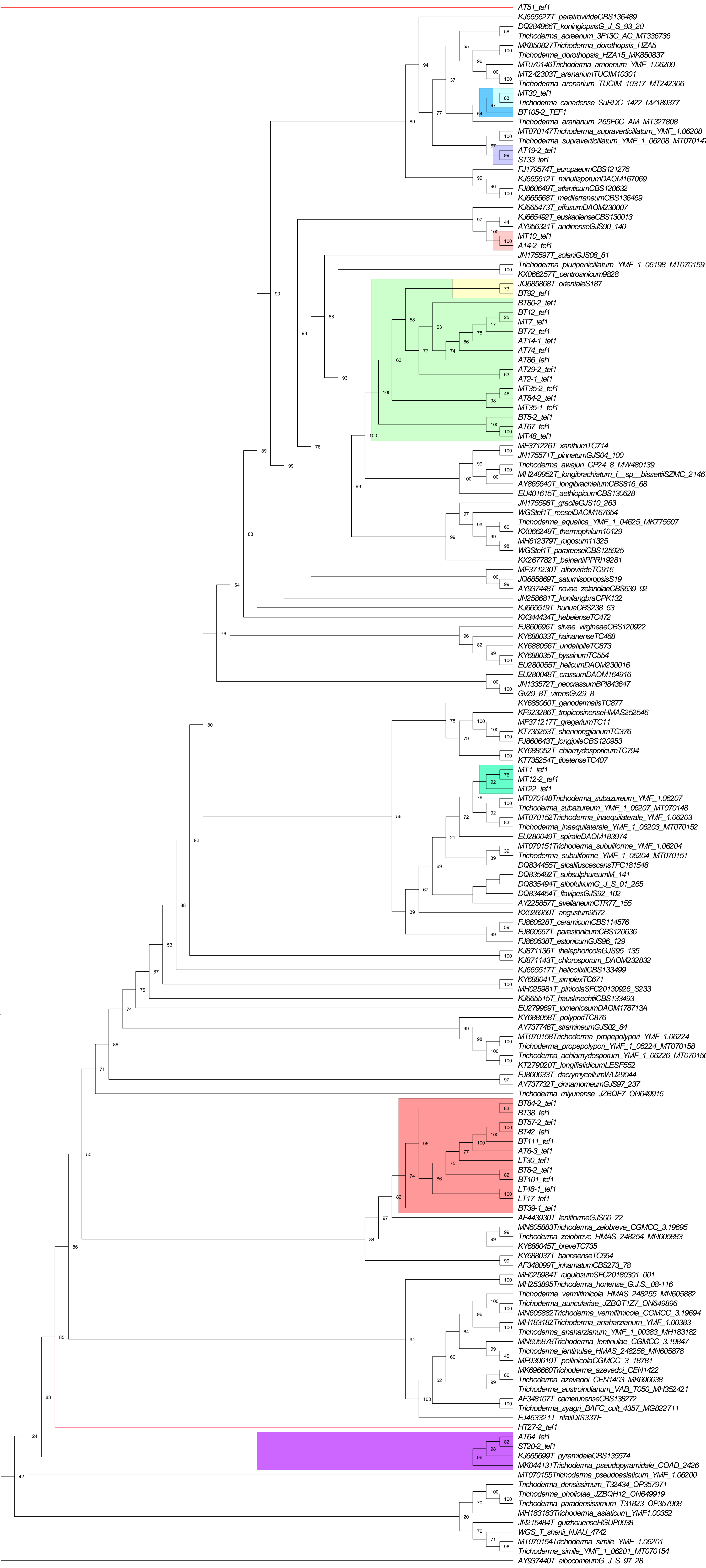

Supplement: fnaf142_Supplemental_Files [file fnaf142_supplemental_files.zip › Supplementary Figure 1.pdf]
